# Demonstrating the Effectiveness and Economic Value of EBPs: Fall Prevention and CDSME Programs

**DOI:** 10.1093/geroni/igaf122.4373

**Published:** 2025-12-31

**Authors:** Brian Ezeonu, Reena Sethi, Kathryn Zahm

**Affiliations:** National Council on Aging, Arlington, Virginia, United States; National Council on Aging, Arlington, Virginia, United States; National Council on Aging, Arlington, Virginia, United States

## Abstract

Two growing challenges, falls and chronic diseases, are among the costliest and most debilitating for older adults. Falls are the leading cause of fatal and non-fatal injuries among adults aged 65 and older and 60-75% of older adults are living with multiple chronic conditions in the United States, both risk factors combined lead to the highest health care expenditures for adults aged 65 years and older. With one in five Americans projected to be aged 65 years or older by 2030 the demand for cost-effective strategies to prevent falls and manage chronic diseases is more urgent than ever. This study uses pre-post data drawn from NCOA’s Healthy Aging Programs Integrated Database (HAPID), covering over 800,000 participants that enrolled in ACL-funded fall prevention and chronic disease self-management education (CDSME) programs to analyze evidence-based fall prevention and CDSME program effectiveness and economic value. Fixed effects and random effects regression models demonstrate that both programs significantly improve health outcomes such as reduced falls, enhanced self-rated health, lower loneliness, and improved self-efficacy. The ROI analysis for fall prevention using avoided healthcare utilization incidents per participant and literature-based healthcare cost estimates shows economic returns ranging from $8.36 to $38.04 per dollar invested. CDSME results showed a 6% increase in self-rated health, 4% improvement in self-efficacy, and up to $153 million in mental health cost savings. Findings from both studies show that evidence-based falls prevention and CDSME programs are not only effective, but fiscally sound.

